# A deep learning-driven cataract screening model derived from multicenter real-world dataset

**DOI:** 10.3389/fmed.2025.1691419

**Published:** 2025-11-27

**Authors:** Zhonghui Cui, Yu Cheng, Siqi Pan, Yong Zhu, Weiwei Dai

**Affiliations:** 1 Department of Ophthalmology, The Second Affiliated Hospital of Anhui Medical University, Hefei, China; 2 Aier Academy of Ophthalmology, Central South University, Changsha, China; 3 Institute of Digital Ophthalmology and Visual Science, Changsha Aier Eye Hospital, Changsha, China; 4 Anhui Aier Eye Hospital, Anhui Medical University, Hefei, China

**Keywords:** deep learning, cataract diagnosis, multicenter data, real-world study, medical image analysis

## Abstract

**Introduction:**

Cataracts, the leading cause of reversible blindness globally, require timely detection and intervention for effective prevention of blindness. Artificial intelligence can assist in massive screening, however, existing models often trained on homogeneous, single-center data, suer from poor generalizability.

**Methods:**

To address this challenge, we developed and validated a deep learning model trained on a large-scale, multicenter, real-world dataset comprising 22,094 slit-lamp images from 21 ophthalmic institutions across 12 provinces and municipalities in China. We designed a cascaded framework that emulates the sequential reasoning of a clinical diagnostic workflow, a methodological approach for ensuring reliability on noisy, real-world data. It first performs an automated quality assessment, then screens for common confounders like pterygium, and finally conducts a differential diagnosis among cataract, post-cataract surgery, other ocular diseases, and healthy eyes. Within this framework, we evaluated several deep learning architectures.

**Results:**

In the cataract classification task, the leading models demonstrated excellent performance on an independent test set. For instance, the ResNet50-IBN based model achieved an accuracy of 93.74%, specificity of 97.74% and an area under the curve (AUC) of 95.30%.

**Discussion:**

This study demonstrates that training on multicenter, real-world data yields a robust and generalizable model, providing a powerful tool for largescale ophthalmic screening. Specifically, our model establishes a methodological blueprint for developing trustworthy medical deep learning systems.

## 1 Introduction

Cataract, as the leading cause of reversible blindness worldwide, poses a serious threat to human visual health (1). According to the statistics of the World Health Organization, approximately 94 million individuals suffered from cataract in 2019 (2), a figure projected to grow with the aging global population. Age is the key factor in the onset of cataracts, and almost all elderly people will be afflicted by this disease (3). Epidemiological studies have consistently indicated a higher prevalence and earlier onset of senile cataracts in Asian populations compared to their European counterparts, highlighting specific demographic vulnerabilities (4). This demographic disparity underscores the urgent need for scalable and accessible screening solutions, particularly in high-prevalence regions.

The standard diagnostic approach for cataracts involves slit-lamp biomicroscopy by ophthalmologists, with grading based on validated standards such as the Lens Opacities Classification System II (LOCS II) (5). However, this experience-dependent paradigm has significant limitations. For instance, inter-observer agreement among physicians with varying experience is often inconsistent, which undermines diagnostic reproducibility (6). This challenge is particularly evident when identifying and grading subtle cataract variants, such as posterior subcapsular or cortical opacities, often leading to diagnostic inconsistencies (7). These limitations are not merely academic; they directly affect clinical outcomes and patient management decisions (8). Moreover, this challenge is exacerbated in resource-limited settings, where the scarcity of trained ophthalmologists presents a major barrier to timely care (9).

Recent advancements in deep learning algorithms, leveraging their robust capacity for automated feature extraction, has demonstrated distinctive advantages in medical image analysis. Indeed, pioneering studies have shown that deep learning models can automatically detect and grade cataracts from slit-lamp images, achieving performance comparable to, or even exceeding, that of human experts on curated datasets (10–13). Some models have even extended this capability to smartphone-based imaging, showing promise for point-of-care diagnostics (14).

However, a critical limitation of many existing models is their reliance on homogeneous data from single centers or highly controlled clinical trials (15). This approach fails to capture the vast heterogeneity of real-world clinical practice, including variations in imaging equipment, patient populations, and examination procedures. Consequently, when deployed in new clinical settings, the performance of these models often degrades significantly due to “domain shift”—the performance decay that occurs when a model is applied to data from a different source or distribution (e.g., different cameras, patient populations, or clinical settings) than the data it was trained on—a common problem in medical artificial intelligence (AI) that severely limits their clinical applicability and generalizability (16, 17). This gap between algorithmic potential and clinical reality is the primary bottleneck preventing the widespread adoption of AI in ophthalmology.

To bridge this crucial gap between research and real-world application, this multicenter study developed and validated a deep learning cataract screening model based on real-world data (RWD) from 21 ophthalmic institutions across 12 provinces and municipalities in China. To mirror the diagnostic process of ophthalmologists and handle the complexities of real-world data, we developed a cascaded framework that emulates the clinical workflow. This sequential triage process—assessing image usability before screening for confounders and making a final diagnosis—is not merely a data preparation step but a core methodological choice designed to enhance the model's robustness and trustworthiness in practical screening scenarios. By systematically training and evaluating multiple advanced deep learning models on this large-scale, diverse dataset, this study aims to identify a robust and generalizable solution for effective, large-scale ophthalmic screening.

## 2 Method

### 2.1 Dataset preparation

**Figure 1:**
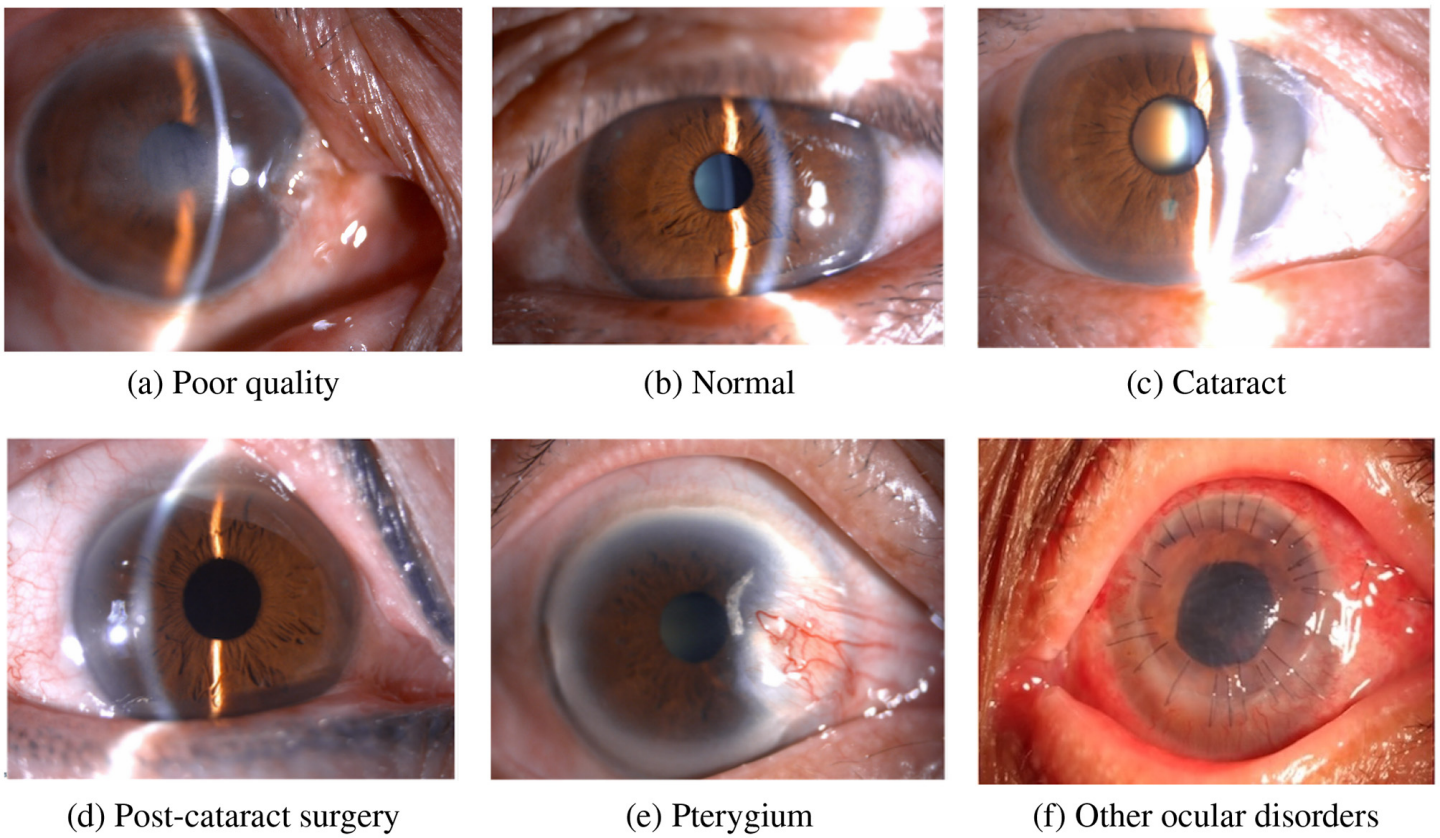
Schematic of annotation categories. **(a)** Poor quality: representing images with low-quality factors. **(b)** Normal: showing a normal ocular condition. **(c)** Cataract: indicating the presence of a cataract. **(d)** Post-cataract surgery: depicting the condition after cataract surgery. **(e)** Pterygium: demonstrating the pterygium disorder. **(f)** Other ocular disorders: referring to other eye-related diseases.

This multicenter study involved 22,094 slit-lamp images sourced from 21 ophthalmic institutions across 12 provinces and municipalities in China: Chongqing, Fujian, Guangdong, Hubei, Hunan, Jiangxi, Liaoning, Ningxia, Shandong, Shanghai, Shanxi, and Yunnan. Images were acquired with standardized digital slit-lamp microscopes (e.g., Topcon SL-D7) between January and February 2024. Two medical image graders, each with five years of experience, independently annotated all images. Figure 1 illustrates representative images for each classification category. The criteria for the classification and grading of cataracts in this study include: the LOCS II classification for cortical (C1–C5) and posterior subcapsular cataracts (P1–P4), and the Emery nuclear sclerosis grading scale for nuclear cataracts (N1–N5). Discrepancies between graders were resolved via consensus discussion and adjudication by a third senior expert.

The image quality assessment step was framed as a binary classification task designed to automatically filter out clinically uninterpretable images. Two experienced graders labeled each image as either “high quality” or “poor quality” based on a standardized protocol. An image was classified as “poor quality” if it exhibited one or more of the following common defects: Improper positioning: the pupil was not centrally located, or the eye was not properly aligned. Inadequate Exposure: The image was either overexposed (too bright, loss of detail) or underexposed (too dark, features obscured). Poor focus: critical structures for diagnosis, such as the iris or anterior lens surface, were blurry. Incorrect slit-lamp illumination: the slit beam was too wide, too narrow, or improperly positioned, failing to illuminate the target structure effectively. Incomplete view: the cornea was not fully visible, or significant portions were obscured by eyelid shadows. Missing illumination: the background light was off, or the slit beam was absent when required for lens assessment. Images that met the criteria for proper position, focus, exposure, and illumination were labeled “high quality.” This annotated dataset was then used to train the image quality assessment model to replicate the expert assessment process.

For the binary classification task of pterygium identification, a balanced subset of 3,783 images was created. This subset comprised all 2,034 images of pterygium cases, along with 1,749 images randomly sampled from the other non-pterygium classes (cataract, post-cataract surgery, other ocular diseases, and normal). This balancing was critical because non-pterygium images significantly outnumbered pterygium cases in the high-quality dataset. Training on such an imbalanced dataset could lead to a model biased toward the majority class, resulting in poor sensitivity for detecting actual pterygium. By creating a balanced subset, we ensured the model would learn the distinctive features of pterygium effectively and minimize false negatives.

Prior to model training, all slit-lamp images underwent a standardized preprocessing pipeline comprising three sequential stages: (1) Spatial standardization via bicubic interpolation to 224 × 224 pixels, preserving morphological integrity while aligning with ResNet architecture requirements; (2) Photometric normalization linearly scaling pixel intensities to [0, 1] to mitigate inter-device variability between Haag-Streit (12-bit) and Topcon (8-bit) systems; (3) Stochastic augmentation incorporating axial rotation ($\theta \sim U\left[-15^{•},+15^{•}\right]$), mirror flipping (*p*_horizontal/vertical_ = 0.5), and photometric perturbation ($\Delta_{\text{brightness}}/\Delta_{\text{contrast}}\sim U\left[-20\%,+20\%\right]$). The augmented dataset was stratified into training/validation/test subsets (8:1:1) with category distribution consistency validated by Kullback-Leibler divergence (KL < 0.01), ensuring balanced representation for parameter optimization (training), early stopping (validation), and unbiased performance assessment (test). In addition, the dataset was split at the patient level to ensure that images from the same patient did not appear in both the training and test sets.

### 2.2 Algorithm selection

This study employed the ResNet50-IBN and the BiSeNet for comparative evaluation in classification tasks, with both architectures being deeply optimized to address the challenges of classifying images with small localized lesions against complex backgrounds in ocular surface images. The ResNet50-IBN architecture builds upon the classical ResNet50 framework (16). It incorporates an IBN module that combines IN's domain adaptation capability with BN's generalization performance. This enhanced architecture maintains the four-stage hierarchical feature extraction of ResNet50 while effectively mitigating domain variations caused by complex background interferences such as specular reflections, eyelashes, and pupillary regions. The Bilateral Segmentation Network (BiSeNet) adopts a dual-branch architecture, comprising a detail branch with shallow, high-resolution convolutions to preserve detailed image information, and a semantic branch that captures global context through deep convolutions (18). These two branches, with the help of the feature fusion module, achieve the integration of multi-scale information. BiSeNet is a lightweight architecture that only has 12.8 million parameters, with the number of floating-point operations being less than 30 billion, resulting in a relatively low computational cost. In addition to these two models, we also included several other well-established architectures in our comparative analysis to ensure a comprehensive evaluation. These included DenseNet-121, known for its feature reuse capabilities (19); EfficientNet-B3, which systematically scales model depth, width, and resolution (20); and ConvNeXt-Tiny, a modern convolutional architecture inspired by Vision Transformers (21). This selection allowed us to assess a diverse range of architectural philosophies on the cataract classification task.

### 2.3 Model development

This study used a deep learning framework to develop a cataract screening model. To balance GPU memory utilization and gradient update stability, a batch size of 16 was adopted. To reduce overfitting risks, the AdamW optimizer was employed. This optimizer combines adaptive learning rate adjustment with weight decay regularization. As the optimization objective, a standard cross-entropy loss function was selected. This methodological choice was deliberate, aiming to train the model on the natural data distribution to mirror the real-world prevalence of conditions encountered in clinical practice. The goal was to evaluate performance under conditions that closely approximate the eventual deployment scenario. The initial learning rate was set at 1 × 10^−4^.

A polynomial decay schedule is used to govern learning rate adjustments throughout the 200 - epoch training span. A minimum learning rate threshold of 5 × 10^−6^ is established to stabilize fine-tuning in the later training phases. A linear warm-up phase spanning the initial 20 epochs was incorporated. The learning rate is progressively scaled from 1% of the initial value to the nominal rate, which mitigates gradient instability during parameter initialization. The complete training regimen encompassed 200 epochs to ensure model convergence. All experiments were conducted with the PyTorch framework on computational platforms equipped with NVIDIA A800 GPUs.

### 2.4 Statistical analysis

To comprehensively evaluate the model's performance, multiple evaluation metrics are employed, including accuracy, sensitivity (recall), specificity, F1-score, and the area under the receiver operating characteristic curve (AUC-ROC). Performance metrics, including accuracy, sensitivity, specificity, F1-score, and AUC, were calculated using the Python scikit-learn library (https://scikit-learn.org/stable/_sources/about.rst.txt). The receiver operating characteristic (ROC) curves were then visualized using TensorBoard (https://www.tensorflow.org/tensorboard). The performance metrics, namely true positive (TP), true negative (TN), false positive (FP), and false negative (FN), are calculated using the following formulas:


$$\begin{array}{l} \text{Sensitivity}=\frac{\text{TP}}{\text{TP}+\text{FN}},\;\text{Specificity}=\frac{\text{TN}}{\text{TN}+\text{FP}},\\ \text{Accuracy}=\frac{\text{TP}+\text{TN}}{\text{TP}+\text{TN}+\text{FP}+\text{FN}} \end{array}$$ (1)

The F1-score was derived from precision (Precision) and recall (Recall), defined respectively as:


$$\begin{array}{l} \text{Precision}=\frac{\text{TP}}{\text{TP}+\text{FP}},\;\text{Recall}=\frac{\text{TP}}{\text{TP}+\text{FN}} \end{array}$$ (2)

The F1-score, calculated as their harmonic mean, is given by:


$$\begin{array}{l} F_{1}=\frac{2\times \text{Precision}\times \text{Recall}}{\text{Precision}+\text{Recall}} \end{array}$$ (3)

We adopted the bootstrapping method to estimate the 95% confidence intervals (CIs) of the performance metrics. To determine if the performance differences between the top-performing models were statistically significant, pairwise McNemar's tests with Yates' continuity correction were conducted. A corrected p-value of less than 0.05 was considered to indicate a statistically significant difference.

## 3 Result

### 3.1 Dataset demographics

**Figure 2:**
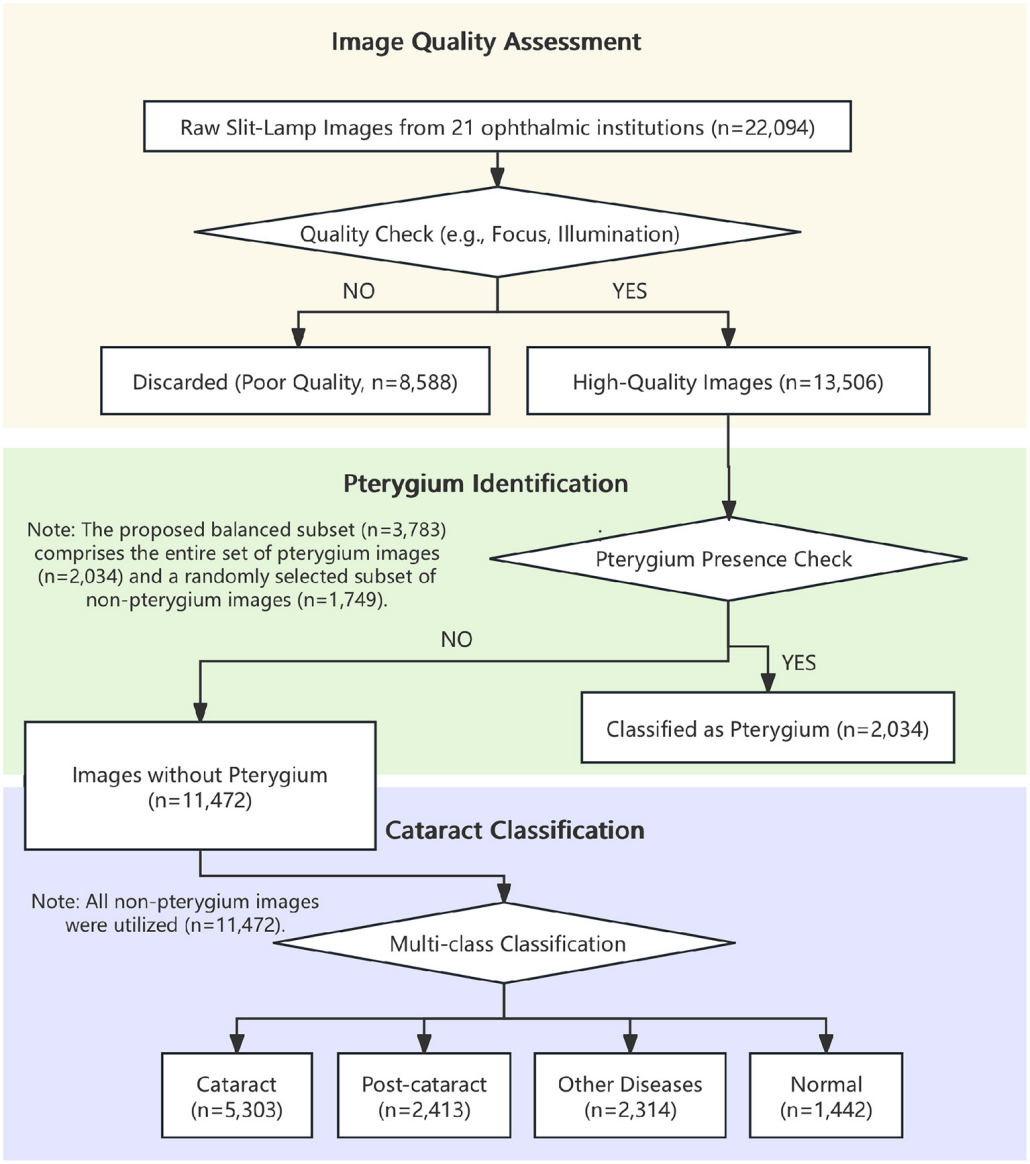
Slit-lamp images analysis flowchart.

The study utilized a comprehensive dataset of slit-lamp images collected from multiple centers. Following the initial data collection, the images were processed through our cascaded framework (Figure 2). The initial step of image quality assessment identified and excluded 8,588 images of poor quality, resulting in a high-quality dataset of 13,506 images for further analysis. An analysis of the excluded images revealed that the primary reasons for poor quality were improper focus, inadequate illumination, and significant motion artifacts, highlighting the practical challenges of data acquisition in real-world clinical settings. Subsequently, the pterygium identification module was applied to this curated dataset, identifying and segregating 2,034 images exhibiting pterygium. The remaining 11,472 images were then used for the final diagnostic classification task, categorized as: cataract (*n* = 5,303), post-cataract surgery (*n* = 2,413), other ocular diseases (*n* = 2,314), and normal (*n* = 1,442). The “other ocular diseases” category was a heterogeneous group designed to test the model's specificity against common confounders and primarily included conditions identifiable on slit-lamp images, such as corneal opacity, signs of uveitis, and subconjunctival hemorrhage. A detailed breakdown of the image distribution across the training, validation, and testing cohorts for each task is provided in Table 1.

**Table 1:** Distribution of training, validation, and test datasets.

**Task**	**Classification**	**Train**	**Validation**	**Test**
Image quality assessment	High quality	10,817	1,468	1,221
	Poor quality	6,826	841	921
	Total	17,643	2,309	2,142
Pterygium identification	Pterygium	1,641	322	71
	Without pterygium	1,422	275	52
	Total	3,063	597	123
Cataract classification	Cataract	4,242	530	531
	Post-cataract	1,930	241	242
	Other disease	1,851	231	232
	Normal	1,153	144	145
	Total	9,176	1,146	1,150

### 3.2 Performance evaluation

The performance of the developed deep learning models was systematically evaluated across the three sequential tasks of the cascaded framework: image quality assessment, pterygium identification, and cataract classification. The detailed performance metrics on the validation and independent test datasets are presented in Tables 2, 3, respectively.

**Table 2:** Model performance on validation dataset.

**Task**	**Model**	**Accuracy**	**Sensitivity**	**Specificity**	**F1**	**AUC**
**Image quality assessment**	**ResNet50-IBN**	**87.15% (85.88%–88.34%)**	**83.94% (82.26%–85.40%)**	**83.94% (82.26%–85.40%)**	**85.04% (83.48%–86.38%)**	**83.94% (82.26%–85.40%)**
	BiSeNet	86.99% (85.60%–88.26%)	83.94% (82.31%–85.48%)	83.94% (82.31%–85.48%)	84.91% (83.36%–86.38%)	83.94% (82.31%–85.48%)
**Pterygium Identification**	**ResNet50-IBN**	**97.82% (96.65%–98.83%)**	**97.74% (96.52%–98.82%)**	**97.74% (96.52%–98.82%)**	**97.81% (96.60%–98.82%)**	**97.74% (96.52%–98.82%)**
	BiSeNet	97.15% (95.81%–98.49%)	97.02% (95.56%–98.29%)	97.02% (95.56%–98.29%)	97.13% (95.74%–98.45%)	97.02% (95.56%–98.29%)
**Cataract classification**	**ResNet50-IBN**	**94.24% (92.76%–95.64%)**	**93.36% (91.45%–95.07%)**	**97.92% (97.36%–98.43%)**	**93.12% (91.29%–94.74%)**	**95.64% (94.43%–96.75%)**
	BiSeNet	94.07% (92.67%–95.38%)	91.92% (89.99%–93.74%)	97.67% (97.11%–98.17%)	92.74% (91.05%–94.36%)	94.80% (93.56%– 95.93%)
	EfficientNet-B3	93.19% (91.71%–94.42%)	92.31% (90.54%–93.95%)	97.59% (97.06%–98.05%)	91.88% (90.10%–93.39%)	94.95% (93.80%– 95.98%)
	DenseNet-121	93.46% (91.97%–94.77%)	91.76% (89.73%–93.55%)	97.57% (97.00%–98.09%)	92.03% (90.14%–93.70%)	94.67% (93.40%– 95.82%)
	ConvNeXt-Tiny	94.42% (93.02%–95.64%)	93.00% (91.06%–94.59%)	97.90% (97.36%–98.36%)	93.30% (91.67%–94.76%)	95.45% (94.22%– 96.48%)

**Table 3:** Model performance on test dataset.

**Task**	**Model**	**Accuracy**	**Sensitivity**	**Specificity**	**F1**	**AUC**
**Image quality assessment**	ResNet50-IBN	88.43% (87.07%–89.88%)	87.25% (85.68%–88.77%)	87.25% (85.68%–88.77%)	87.79% (86.33%–89.29%)	87.25% (85.68%—88.77%)
	**BiSeNet**	**88.52% (87.20%–89.79%)**	**87.30% (85.87%–88.71%)**	**87.30% (85.87%–88.71%)**	**87.87% (86.47%–89.19%)**	**87.30% (85.87%–88.71%)**
**Pterygium identification**	**ResNet50-IBN**	**96.75% (93.50%–99.19%)**	**96.41% (92.79%–99.30%)**	**96.41% (92.79%–99.30%)**	**96.65% (93.21%–99.19%)**	**96.41% (92.79%–99.30%)**
	BiSeNet	95.93% (91.87%–99.19%)	95.45% (91.14%–98.94%)	95.45% (91.14%–98.94%)	98.80% (91.67%–99.14%)	95.45% (91.14%—98.94%)
**Cataract classification**	ResNet50-IBN	93.74% (92.26%–95.04%)	92.86% (90.99%–95.56%)	97.74% (97.18%–98.23%)	92.64% (90.81%–94.18%)	95.30% (94.09%—96.41%)
	BiSeNet	92.35% (90.78%–93.91%)	90.07% (88.10%–92.14%)	97.10% (96.49%–97.71%)	90.71% (88.81%–92.68%)	93.58% (92.30%—94.91%)
	EfficientNet-B3	93.48% (92.09%–94.96%)	92.47% (90.67%–94.31%)	97.61% (97.09%–98.16%)	92.35% (90.66%–94.08%)	95.04% (93.91%—96.22%)
	DenseNet-121	93.39% (91.83%–94.87%)	91.98% (90.13%–93.93%)	97.55% (96.98%–98.09%)	92.15% (90.29%–93.90%)	94.77% (93.57%—96.02%)
	**ConvNeXt-tiny**	**94.42% (93.02%–95.72%)**	**93.00% (91.08%–94.73%)**	**97.90% (97.36%–98.37%)**	**93.30% (91.59%–94.80%)**	**95.45% (94.23%–96.54%)**

In the image quality assessment task, both ResNet50-IBN and BiSeNet performed comparably on the validation set. On the test set, both models showed excellent generalization, achieving high accuracy (ResNet50-IBN: 88.43%; BiSeNet: 88.52%), demonstrating their capability to reliably filter out unusable images.

For the pterygium identification task, both models again achieved highly proficient results on the validation dataset. On the independent test set, both models maintained their strong performance, with ResNet50-IBN achieving a slightly higher accuracy of 96.75% and an AUC of 96.41%, indicating robust performance in identifying this key confounder.

In the task of cataract classification, all models demonstrated well performance on the test dataset (Table 3). Among them, the ConvNeXt-Tiny and ResNet50-IBN models achieved the leading performance metrics. Specifically, ConvNeXt-Tiny obtained an accuracy of 94.42% and an AUC of 95.45%. The ResNet50-IBN model also performed robustly, with an accuracy of 93.74% and an AUC of 95.30%. The performance of other models was also competitive; for instance, DenseNet-121 recorded an accuracy of 93.39%, while EfficientNet-B3 reached 93.48%, demonstrating the effectiveness of these established architectures in handling complex medical images. In contrast, the BiSeNet model showed a slightly lower accuracy of 92.35% on this more challenging classification task, which may be attributed to its lightweight network structure.

**Figure 3:**
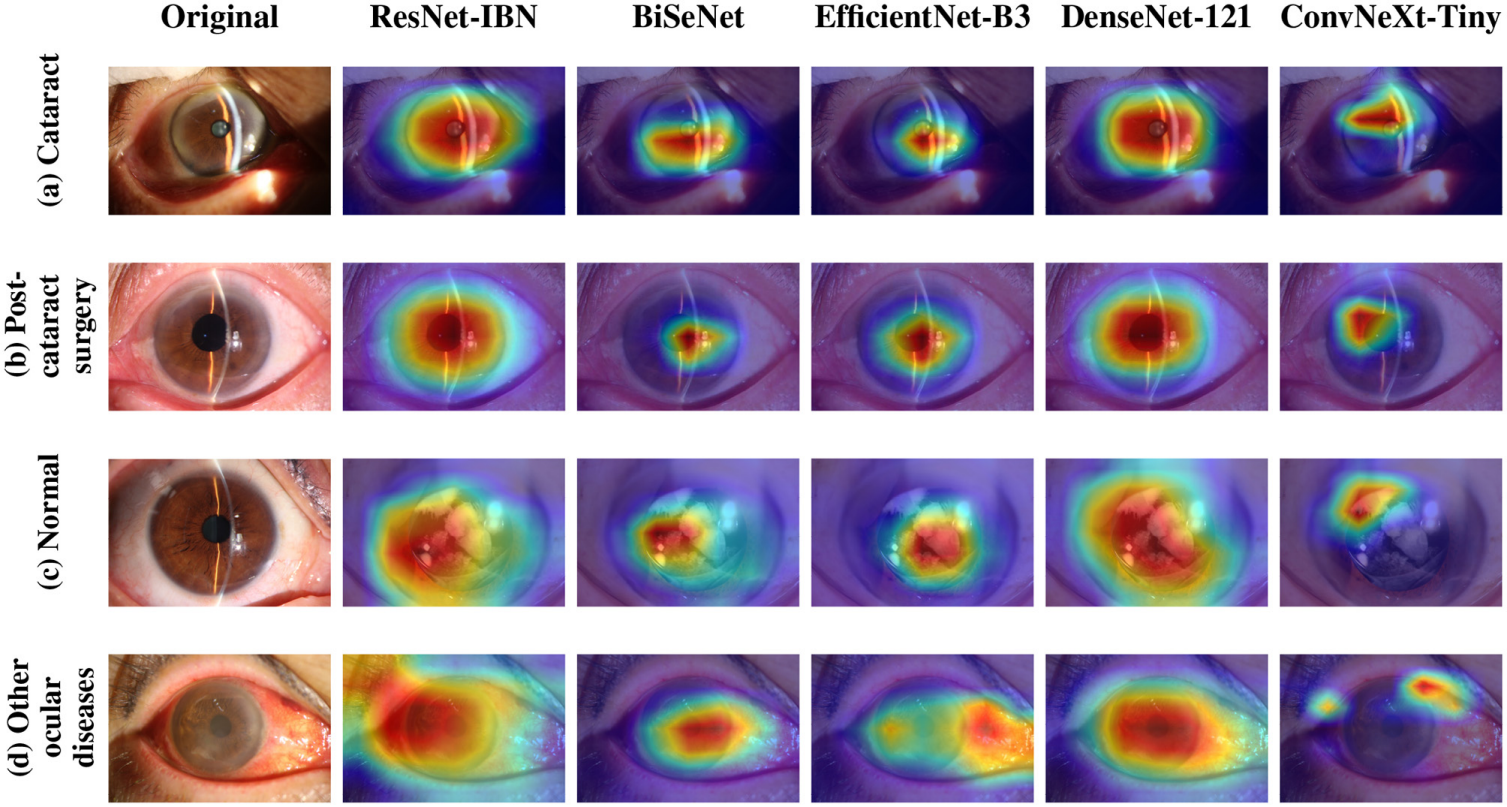
Grad-CAM visualizations of different deep learning models. Each row represents a distinct ocular condition, and each column corresponds to a specific model's attention heatmap. This comparison highlights differences in how models focus on clinically relevant features. **(a)** Cataract. **(b)** Post- cataract surgery. **(c)** Normal. **(d)** Other ocular diseases.

To further investigate the models' decision-making processes, we analyzed their areas of focus using Grad-CAM visualization, as presented in Figure 3. The heatmaps illustrate the image regions that most influenced each model's prediction across four conditions: cataract, post-cataract surgery, normal, and other ocular diseases. A consistent pattern emerged wherein the better-performing models, particularly ResNet50-IBN, showed attention maps that were more precisely localized to clinically relevant features. For instance, in cataract images (row a), the activation area of the ResNet50-IBN model is highly concentrated on the lens opacities, whereas some other models occasionally exhibit more diffuse activation patterns. This precise feature localization likely contributes to its higher diagnostic accuracy.

All leading models maintained high specificity, particularly ResNet50-IBN (97.74%) and ConvNeXt-Tiny (97.90%). This high specificity is of significant clinical value, as it underscores the models' reliability in correctly identifying non-cataract cases, thereby helping to minimize unnecessary referrals resulting from false positives. To determine if these performance differences were statistically significant, pairwise McNemar's tests were conducted. The results, after correction for multiple comparisons, revealed no statistically significant difference between ResNet50-IBN and the other models (p > 0.05 for all comparisons). This suggests that despite minor differences in numerical metrics, all tested deep learning architectures demonstrated strong and comparable performance. Given their similar excellent performance, the choice for a final deployed model could involve trade-offs between computational efficiency (favoring ConvNeXt-Tiny) and architectural robustness for domain shift. For the purpose of this study's subsequent analyses, we focused on ResNet50-IBN, as its superior feature localization, visualized through Grad-CAM (Figure 3), provided clearer insights into the model's decision-making process.

### 3.3 Model generalizability across different devices

A key objective of this study was to develop a model robust to the “domain shift” inherent in multicenter data. The dataset was sourced from various slit-lamp devices, with the distribution detailed in Table 4. To explicitly test the model's generalizability, the performance of the superior ResNet50-IBN model was evaluated on device-specific subgroups within the test set. As detailed in Table 5, the model demonstrated consistently high accuracy and AUC scores across all major device types. For example, it achieved high AUCs on both the most frequent device, Shangbang LS-7 (95.20%), and the TOPCON SL-D701 (93.00%), validating its robustness to device-specific variations.

**Table 4:** Distribution of the Dataset by device source in cataract classification task.

**Device source**	**Training set**	**Validation set**	**Test set**
Shangbang LS-7	4,107 (44.76%)	496 (43.28%)	350 (30.43%)
TOPCON SL-D701	1,440 (15.69%)	187 (16.32%)	167 (14.52%)
TOPCON SL-D7	1,262 (13.75%)	161 (14.05%)	154 (13.39%)
Shangbang LS-6	1,164 (12.69%)	151 (13.18%)	152 (13.22%)
Ruiyu SLM-KD4	411 (4.48%)	50 (4.36%)	46 (4.00%)
Kanghua SLM-4ER	292 (3.18%)	29 (2.53%)	36 (3.13%)
Shangbang LS-7DS	236 (2.57%)	26 (2.27%)	21 (1.83%)

**Table 5:** Performance metrics of the model on different subgroups.

**Subgroup ID**	**Samples**	**Accuracy**	**Sensitivity**	**Specificity**	**F1 Score**	**AUC**
Shangbang LS-7	550	96.00% (94.18%–97.45%)	91.96% (87.35%–96.21%)	98.44% (97.75%–99.04%)	91.71% (87.23%–95.09%)	95.20% (92.63%–97.55%)
TOPCON SL-D701	167	93.41% (89.21%–96.41%)	88.45% (80.78%–95.38%)	97.54% (95.83%–98.72%)	90.14% (82.25%–95.84%)	93.00% (88.52%–96.91%)
Shangbang LS-6	160	92.50% (88.12%–96.25%)	91.89% (87.43%–95.97%)	97.34% (95.84%–98.67%)	92.26% (87.98%–95.99%)	94.62% (91.68%–97.30%)
TOPCON SL-D7	154	91.56% (87.01%–95.45%)	93.44% (89.51%–96.74%)	96.81% (94.98%–98.37%)	93.35% (89.18%–96.70%)	95.13% (92.36%–97.51%)

## 4 Discussion

This study introduces a deep learning framework whose core innovation is its emulation of the clinical diagnostic workflow. This clinically-inspired, hierarchical system (Quality Assessment → Confounder Identification → Differential Diagnosis) yielded several principal findings. Firstly, it demonstrated a robust method for navigating large-scale, real-world data, automatically filtering 38.87% of low-quality images and identifying 9.21% as pterygium cases. This stringent curation is not merely a preprocessing step but a significant finding in itself; the high exclusion rate underscores the critical gap between idealized datasets and routine clinical practice, highlighting the need for standardized image acquisition. Secondly, the resulting model exhibited exceptional diagnostic performance, with top architectures achieving accuracies over 93.74% and AUCs over 95.30%. Our selected ResNet50-IBN model attained a specificity of 97.74%, substantiating its reliability for clinical support. Finally, the framework's ability to differentiate among five clinical conditions provides a more comprehensive and clinically useful assessment than simple binary classifiers, such as the one described in Fung et al. (10).

The framework's demonstrated success is rooted in two strategic choices: its reliance on uncurated, multi-center data and its strict adherence to a logical clinical sequence. Unlike models trained on sanitized datasets, our approach utilized raw images from 21 diverse medical centers, exposing the model to the full spectrum of real-world complexities like eye malposition, motion artifacts, and defocus blur. This direct exposure is central to achieving its strong generalizability. Furthermore, the system mirrors clinical reasoning by addressing challenges in a layered manner. It first establishes technical validity (image quality) before proceeding to anatomical confounders (pterygium) and finally pathological classification. This sequential strategy, which prevents technical artifacts from corrupting clinical interpretation, is fundamental to the system's overall efficacy and trustworthiness.

The systematic design of our study and its effective handling of clinical complexities provide it with distinct advantages over existing ophthalmic AI research. Prior studies have typically focused on single tasks or relied on idealized data. For instance, while Slit-Lamp-GPT integrates a multimodal large language model for report generation, its cataract identification accuracy was only 0.82, and its data screening criteria were not explicitly reported, limiting its direct applicability in real-world screening scenarios (22). Another advanced model, TKD-Net, enhanced the grading accuracy of cortical cataracts through a region decomposition strategy; however, its design did not account for the co-existence of multiple pathologies (e.g., pterygium with co-occurring cataract), a common clinical challenge (23). Our study's cascaded design is the first to integrate quality control, exclusion of confounding lesions, and disease diagnosis into an end-to-end workflow, fundamentally improving diagnostic reliability. Concurrently, the ResNet50-IBN architecture effectively mitigates the domain shift problem arising from multi-center, multi-device data through its use of Instance-Batch Normalization. Its high specificity of 97.74% meets the requirements for clinical decision-making.

While numerical metrics placed ResNet50-IBN and ConvNeXt-Tiny as leading models with no statistically significant performance differences, a deeper analysis reveals ResNet50-IBN's superiority for this clinical task. Performance metrics alone do not tell the full story; interpretability analysis provides critical insight. As visualized with Grad-CAM in Figure 3, the ResNet50-IBN model consistently generates highly focused attention maps on the clinically relevant lens opacities. In contrast, other models like BiSeNet often exhibit more diffuse activation patterns. This precise feature localization, which likely leads to its higher diagnostic accuracy, stems from two key architectural advantages. First, its foundation as a deep classification network (ResNet50) is inherently more suitable for identifying the subtle variations in texture and transparency characteristic of cataracts, compared to segmentation-focused architectures like BiSeNet, which excel at defining clear morphological boundaries (e.g., for pterygium). Second, the integration of the Instance-Batch Normalization (IBN) module is crucial for this multicenter study. This design specifically addresses the domain shift problem introduced by diverse imaging systems from our 21 medical institutions. It enables the model to focus on the pathological features themselves rather than variations in imaging style, thereby improving diagnostic robustness on real-world data. Therefore, ResNet50-IBN is selected not just for its strong performance, but for its more clinically plausible decision-making process and an architecture fundamentally tailored to the challenges of multicenter cataract screening.

A key strength of this study—and a primary challenge for AI in medicine—is the use of a large-scale, multicenter dataset. This diversity inevitably introduces a “domain shift” caused by different imaging equipment. Our explicit testing on device-specific subgroups (Table 5) directly addresses this challenge. The findings that our model maintained consistently high performance across various devices are highly significant. This demonstrates that the model learned true pathological features rather than device-specific artifacts, a crucial step toward clinical translation. This robustness can be largely attributed to the ResNet50-IBN architecture, which is specifically designed to mitigate domain variations. Consequently, the model shows strong potential as a reliable and deployable tool in real-world clinical settings, where a variety of equipment is the norm.

Despite the model's strong performance, several limitations must be acknowledged. First, the geographic and ethnic homogeneity of the dataset is a primary limitation. Although multi-source, the data were collected exclusively from a Chinese population, which may affect the model's generalizability to other ethnic groups globally. Future work should aim to incorporate more diverse international datasets through privacy-preserving techniques such as federated learning (24) to build a more inclusive and robust global model. Second, data quality posed a significant challenge. Our framework excluded 38.87% of images due to poor quality. While this strict filtering step was crucial for ensuring the reliability of the final diagnosis, it also highlights the practical challenges of real-world data acquisition. Third, our choice to forgo class weighting in favor of training on the natural data distribution, while intended to reflect clinical reality, is a limitation worth noting. This approach may lead to reduced sensitivity for underrepresented diagnostic categories. Future work could systematically explore the impact of class balancing techniques to determine if sensitivity for minority classes can be improved without compromising the model's robust performance on the more prevalent conditions. Finally, the model's diagnostic depth and interpretability have room for improvement. The current model identifies the presence of cataracts but cannot automatically differentiate between its subtypes (e.g., cortical, nuclear) or perform fine-grained grading. Furthermore, it operates as a “black box,” and the biological logic behind its decisions remains unclear. Future work could incorporate specific strategies, such as the region decomposition used in TKD-Net (23), to achieve more fine-grained automated grading. Additionally, leveraging attention mechanisms to correlate model-focused image features with known biological markers—such as oxidative stress in lens epithelial cells (25) or cellular senescence (26)—could enhance the model's interpretability and clinical trust. Lastly, for practical deployment, particularly in large-scale community screening, model efficiency is a critical consideration. The current architectures, while powerful, may require significant computational resources. Future research should focus on model compression and optimization techniques—such as knowledge distillation, quantization, or pruning—to develop lightweight versions that can be deployed on more accessible, resource-constrained devices like smartphones or edge computing platforms without a significant loss in performance.

Looking ahead, a clear path toward clinical translation will be the focus of this subsequent work. This includes: (1) conducting prospective clinical trials to validate the model's real-world impact on patient referral accuracy and efficiency; (2) integrating the system into existing Electronic Health Record (EHR) platforms and teleophthalmology networks to streamline its application; and (3) developing a user-friendly interface for deployment on mobile or portable devices for community-based screening. Through these efforts, we aim to translate AI innovation into tangible clinical tools.

## 5 Conclusions

This study demonstrates that a deep learning framework, designed to emulate clinical reasoning, can achieve robust and generalizable performance for cataract screening using a large-scale, multicenter, real-world dataset. The high diagnostic accuracy and specificity of our model validate its potential as a powerful tool for large-scale ophthalmic screening and establish a methodological blueprint for developing trustworthy medical AI systems from real-world evidence.

## Data Availability

The raw data supporting the conclusions of this article will be made available by the authors, without undue reservation.
